# Editorial: Precision therapy and biomarkers in head and neck squamous cell carcinoma

**DOI:** 10.3389/fonc.2024.1442630

**Published:** 2024-07-08

**Authors:** Luis Abel Quiñones, Fujun Han, Ye Guo

**Affiliations:** ^1^ Laboratory of Chemical Carcinogenesis and Pharmacogenetics (CQF), Department of Basic and Clinical Oncology, Faculty of Medicine, University of Chile, Santiago, Chile; ^2^ Department of Pharmaceutical Sciences and Technology, Faculty of Chemical and Pharmaceutical Sciences, University of Chile, Santiago, Chile; ^3^ Latin American Network for Implementation and Validation of Clinical Pharmacogenomics Guidelines (RELIVAF), Santiago, Chile; ^4^ Cancer Center, First Hospital of Jilin University, Changchun, China; ^5^ Department of Oncology, Shanghai East Hospital, School of Medicine, Tongji University, Shanghai, China

**Keywords:** biomarker, epidermal growth factor receptor, head and neck squamous cell carcinoma, immune checkpoint inhibitor, precision therapy

Head and neck squamous cell carcinoma (HNSCC) ranks as the seven most prevalent cancer worldwide with over half of patients presenting with locally advanced disease. Its incidence and mortality rates display substantial variation across different populations, depending on sex, age and socioeconomic status. Despite curative multimodal therapy, a significant proportion will experience recurrence or distant metastasis (1). Prognosis for recurrent or metastatic HNSCC remains poor, with therapeutic options including conventional chemotherapy, epidermal growth factor receptor inhibitors (EGFRis), and immune checkpoint inhibitors (ICIs) (2, 3). Comprehensive genomic profiling identifies distinct HNSCC subtypes for therapeutic stratification (4, 5). Therefore, there is a pressing and urgent need to identify and validate clinically significant biomarkers to improve therapeutic prediction and explore novel drug targets and combination therapies involving ICIs to enhance survival and overcome resistance in HNSCC. In order to achieve that, this Research Topic aimed to underscore the imperative of identifying prognostically significant biomarkers and exploring druggable targets in HNSCC, emphasizing originality, significance, and timeliness. The manuscripts compiled here addressed this purpose from different points of view.

In this context, Liu et al. analysed HNSCC samples using data from The Cancer Genome Atlas and GSE41613 datasets, supplemented with 199 focal adhesion-related genes (FARGs) from the Molecular Signatures database. Focal adhesion, functioning as a connector between tumour cells and the extracellular matrix, plays crucial roles in tumour invasion, migration, as well as therapeutic resistance. Employing cluster analysis, they reduced dataset dimensions and classified patients into subclusters. Their FARG signature model calculated risk scores for each patient, aiding in subgroup quantification, prognostic prediction, immune infiltration status, and therapeutic response evaluation. Results unveiled two HNSCC molecular subtypes, with C2 patients displaying shorter overall survival. The nine-gene FARG signature correlated with decreased overall survival and emerged as an independent prognostic factor. Furthermore, the FARGs were linked to immune invasion, gene mutation status, and chemosensitivity. Additionally, overexpression of MAPK9, one of the nine genes, in HNSCC tissues hindered cell proliferation, migration, and invasion, offering insights for cancer therapies. In another article, Wu et al. investigated the impact of Tribbles Pseudokinase 3 (TRIB3) on HNSCC. TRIB3, a member of pseudokinase protein family, is involved in a diverse range of biological activities, from cell signal transduction and metabolic regulation to stress responses and immune modulation. Utilizing RNA-sequence data from the TCGA database, they analysed TRIB3 expression patterns and assessed its prognostic value in HNSCC patients. The study explored TRIB3’s correlation with tumour mutation burden, clinical data, immune checkpoint genes, and immune cell infiltration. TRIB3’s location in tumour tissues and protein interactions were identified through databases. Gene set enrichment analysis assessed TRIB3 function, while RT-qPCR and immunohistochemistry verified its expression in clinical samples. *In vitro* validation illustrated TRIB3’s role in enhancing HNSCC cell malignancy. The results highlighted significant overexpression of TRIB3 in the nucleus and cytoplasm of HNSCC cells, which enhanced tumour cell migration. While not predictive for the efficacy of ICI treatment, TRIB3 emerged as an independent prognostic factor, correlating with advanced tumour stage, tumour mutation burden, immune cell infiltration, and immune evasion-related genes. In conclusion, TRIB3 could serve as a potential prognostic marker and a pivotal gene in HNSCC immune evasion.

On the other hand, the work of Xue et al. aimed to identify biomarkers and assess the prognostic impact of CDKN2A mutations in a retrospective study of 77 patients with recurrent or metastatic HNSCC, of whom 62 received ICIs, using next-generation sequencing (NGS) data from Foundation Medicine (FM). They compared CDKN2A loss-of-function (LOF) status in relation to wild-type (WT), revealing that CDKN2A alterations indicate poor survival outcomes in HNSCC patients, including those receiving ICIs.

An interesting case report is discussed by Deng et al. involving a 71-year-old Chinese woman who was diagnosed with both lung adenocarcinoma and oesophageal squamous cell carcinoma. The patient exhibited symptoms including swallowing difficulties. The mutational status revealed an EGFR gene mutation (exon 21 L858R) in the lung adenocarcinoma, and the oesophageal carcinoma exhibited EGFR overexpression. Despite the conventional recommendation for surgical intervention, she declined this approach and instead chose to undergo treatment with icotinib, an EGFR tyrosine kinase inhibitor. After a five-year follow-up period, the patient exhibited no signs of recurrence or metastasis, with both cancers remaining stable and the oesophageal lesion nearly cured. The success of icotinib in this oesophageal squamous cell carcinoma case, coupled with the broader approval of EGFRi for treating HNSCC, underscores a crucial point: EGFR pathway involvement can be seen across different squamous cell carcinomas, opening up potential therapeutic avenues.

Finally, Puttagunta et al. deliver a mini-review investigating the present and future potential of vascular endothelial growth factor-tyrosine kinase inhibitors, designed to hinder tumour angiogenesis in HNSCC. They conclude that their combination with immunotherapy shows considerable promise, as exemplified by recent trials like ICIs with lenvatinib or cabozantinib. Moreover, exploring such combination therapies preoperatively in locally advanced disease presents another intriguing avenue for investigation.

Overall, the contents of the articles in this Research Topic further highlight the need to expand research on biomarkers for HNSCC diagnosis, prognosis, and therapeutic options. A better understanding of HNSCC biology is indeed a prerequisite for ensuring the development of targeted and ICI therapies with enhanced efficacy and reduced toxicity (6–8).

## Author contributions

LQ: Writing – original draft, Writing – review & editing. FH: Writing – original draft, Writing – review & editing. YG: Writing – original draft, Writing – review & editing.
